# Seroepidemiology of Hepatitis E Virus Infection in an Urban Population in Zambia: Strong Association With HIV and Environmental Enteropathy

**DOI:** 10.1093/infdis/jit409

**Published:** 2013-08-06

**Authors:** Choolwe Jacobs, Clarance Chiluba, Cynthia Phiri, Mpala Mwanza Lisulo, Mumba Chomba, Philip C. Hill, Samreen Ijaz, Paul Kelly

**Affiliations:** 1 Tropical Gastroenterology and Nutrition Group, UNZA School of Medicine, Lusaka, Zambia; 2 Department of Preventive and Social Medicine, University of Otago, New Zealand; 3 Virology Reference Laboratory, Health Protection Agency, Colindale; 4 Blizard Institute, Barts & The London School of Medicine, Queen Mary, University of London, United Kingdom

**Keywords:** hepatitis E virus, HIV infection, environmental enteropathy, epidemiology, seroprevalence

## Abstract

***Background.*** Hepatitis E virus (HEV) infection causes major epidemics of infectious hepatitis, with high mortality rates in pregnant women. Recent reports indicate that HEV coinfections with human immunodeficiency virus (HIV) may have a more protracted course. However, the impact of HEV infections in communities heavily affected by HIV remains poorly studied. We set out to examine age-related seroprevalence in a community where we have previously carried out studies on environmental enteropathy.

***Methods.*** Blood samples from 194 children and 106 adults were examined for immunoglobulin G and immunoglobulin M antibodies for HEV. HEV data were correlated with HIV status and morphometric analysis of small intestinal biopsies.

***Results.*** Seroprevalence rose throughout childhood, from 8% in children aged 1–4 years, to 36% in children aged 10–14 years. In adults, the overall prevalence was 42%, with 28% in HIV-seronegative adults and 71% in HIV-seropositive adults (odds ratio, 6.2; 95% confidence interval, 2.2–18; *P* = .0001). In adults, villous height and crypt depth measurements showed that HEV seropositivity was associated with worse enteropathy (*P* = .05 and *P* = .005, respectively).

***Conclusions.*** HEV infection is common in Zambia. In adults it is strongly associated with HIV status, and also with environmental enteropathy.

Epidemics of jaundice, characterized by a high mortality rate in pregnant women, have been recorded for >200 years [1]. The causative virus of this form of non-A, non-B hepatitis was recognized in 1983 to be hepatitis E virus (HEV), a nonenveloped, single-stranded RNA virus that has only 1 serotype but 4 major genotypes [2]. It is one of the leading causes of hepatitis globally, and epidemics can be very large with 29 300 cases recorded in Delhi in 1954–1955 [1]. Throughout the developing world, case fatality rates of up to 73% in pregnancy have been recorded, although the highest figures derived from hospital records probably reflect selection bias toward the most severe cases. Incidence rates in the developing world are hard to estimate, but one study in Bangladesh calculated the rate at 60.3 per 1000 person-years [3].

More recently, the development of chronic hepatitis linked to HEV infection has been described in immunosuppressed patients [4–6]. These studies report a persistence of HEV RNA as well as progression of liver inflammation and fibrosis with HEV-related cirrhosis also being described. Such infections were initially reported in the transplant setting but have now also been observed in hematological patients receiving chemotherapy and in patients infected with human immunodeficiency virus (HIV). As areas of high HIV prevalence overlap widely with regions where HEV is common, the potential for coinfection will be high, which may lead to the emergence of comorbidity. This will be of particular significance in the developing world, which bears the brunt of the HIV pandemic. However, there are few data on HEV epidemiology in African populations and this area remains poorly investigated.

Transmission of HEV in the developing world is fecal-oral as with hepatitis A virus, but unlike hepatitis A virus infection, antibody levels wane over time. Whether this reflects imperfect serological tests or a genuine difference in the immune response is uncertain, but there is some evidence that early childhood infection does not protect against reinfection in adulthood [2]. Transmission of HEV through the developing world is via the fecal-oral route linked to contaminated food and water. We have previously reported that tropical enteropathy shows strong evidence of environmental causation, with pronounced seasonal effects and relationships to poor housing and asymptomatic intestinal carriage of pathogens [7]. We now refer to this phenomenon as environmental enteropathy [8], as it is not exclusive to the tropics and there are tropical populations (such as Qatar and Singapore) where it is not found [9].

We set out to investigate the seroepidemiology of HEV infection in both children and adults from a poor township of Lusaka, Zambia. Additional analyses were undertaken to explore associations between HEV and HIV infection and also with environmental enteropathy.

## METHODS

Two separate sample sets were used. To analyze seroprevalence in adults, archived serum samples were used from a community study of intestinal infectious disease in Misisi township [10, 11], which were collected in 1999. The study for which the samples were obtained was considered fully representative of the local community [10]. We have previously published full data on HIV seroprevalence and morphometric analysis of intestinal biopsies from the adults in that community study [7]. Additional data were available on hygiene characteristics of the house, place of upbringing (urban/rural) up to age 15 years, educational achievement, and stool parasitology (Table 1). Permission from the Biomedical Research Ethics Committee of the University of Zambia was obtained to analyze hepatitis viruses in these serum samples (006-04-09). All participants had given informed consent for both studies.

**Table 1.** Demographic Characteristics of Children

Variable	Children (n = 194), No. (%)	OR (95% CI)	*P* Value
Female sex	115 (59)	0.64 (.34–1.2)	.23
Age group			
1–4 y	96 (49)	1.0	
5–9 y	62 (32)	1.93 (.81–4.6)	.20
10–14 y	36 (19)	4.3 (2.0–9.6)	.0003
Water source			
Household tap	0		
Communal tap	194 (100)		
Payment required for water, cost per 20 L			
Zero payment	29 (15)	1.0	
K100 (0.05 USD)	98 (50)	2.7 (.66–10.8)	.16
K200 (0.10 USD)	67 (35)	2.4 (.56–10.1)	.33
Treatment of drinking water by chlorination or boiling			
No	171 (88)		
Yes	23 (12)	0.25 (.04–1.7)	.13
Vessel used for storing water			
Container	71 (37)	0.86 (.43–1.7)	.84
Bucket	122 (63)		
Amount of water stored in home			
≤20 L	52 (27)	1.0	
20–40 L	79 (41)	0.86 (.41–1.8)	.82
≥40 L	63 (32)	0.66 (.28–4.6)	.44
Have own pit latrine			
No	155 (80)		
Yes	39 (20)	0.76 (.31–1.9)	.63
Use soap to wash hands			
No	112 (58)		
Yes	82 (42)	0.65 (.32–1.3)	.24
Wash hands after using the toilet			
No	23 (12)	1.25 (.41–3.8)	1.00
Yes	171 (88)		

Abbreviations: CI, confidence interval; OR, odds ratio; USD, US dollars.

To analyze seroprevalence in children, a prospective study was carried out in 2011 in the same community, and approval was obtained from the same committee (009-06-11). We randomly selected 1 zone (section C) out of 4 using simple random selection. Starting at the home of the “head of the community,” households were then approached outward in all directions from there, until the required number of children (n = 194) was met. This sample represented approximately 40% of the total number of children in zone C. Recruitment of participants within this cluster of closely grouped houses in a well-defined geographical area within the suburb was thought to be fully representative and important to increase probability that residents would share a similar degree of exposure to enteric pathogens [12]. Written informed consent was obtained from willing caregivers and assent was obtained from eligible children >5 years of age. Eligible children were between the ages of 1 and 15 years and residents of the Misisi compound (ie, not visiting). If a child >5 years of age refused assent to participate even if the caregiver had consented, he/she was excluded from the study. Children and caregivers were invited to an urban clinic (10 minutes’ walk) for interviews and blood tests. At the clinic, a structured questionnaire was used to collect demographic data and information about household facilities from caregivers. Blood samples (4 mL) were then collected following Ametop (amethocaine) topical anaesthetic patches applied for 30 minutes prior to venipuncture. Serum was obtained by centrifugation and stored at −80°C for <2 months prior to analysis.

### Virological Analysis

All samples were tested for anti-HEV immunoglobulin G (IgG; Fortress Diagnostics Ltd) by enzyme-linked immunosorbent assay (ELISA). Samples reactive for IgG antibody were further tested for anti-HEV immunoglobulin M (IgM; Fortress Diagnostics Ltd). The ELISA testing was undertaken in accordance with the manufacturer's instructions, with the exception that a higher cutoff was used: samples with a sample to cutoff ratio >1.5 were declared to be seropositive. The sensitivity and specificity of the ELISA assay used in this study were determined by the manufacturer as 99.9% and 99.08%, respectively.

### Data Analysis

Data (demographic, clinical, and serological) were double-entered into Epi Info 6 and then Stata 10.1 software and analyzed using frequencies and percentages. Odds ratios (ORs) with 95% confidence intervals (CIs) were derived and hypothesis testing was done using χ^2^ or Fisher's exact test. Multivariate analysis was carried out using unconditional logistic regression in Stata 12 (Stata Corp).

In Misisi, environmental enteropathy is virtually ubiquitous, so villous height, crypt depth, and epithelial surface area are presented as continuous variables to represent the severity of mucosal damage. Our previous publication [7] sets out details of reproducibility and validation of the morphometric analysis that generates measures of villous height (µm), crypt depth (µm), and epithelial surface area (measured as length [µm] per 100 µm of muscularis mucosae).

## RESULTS

**Figure 1.**
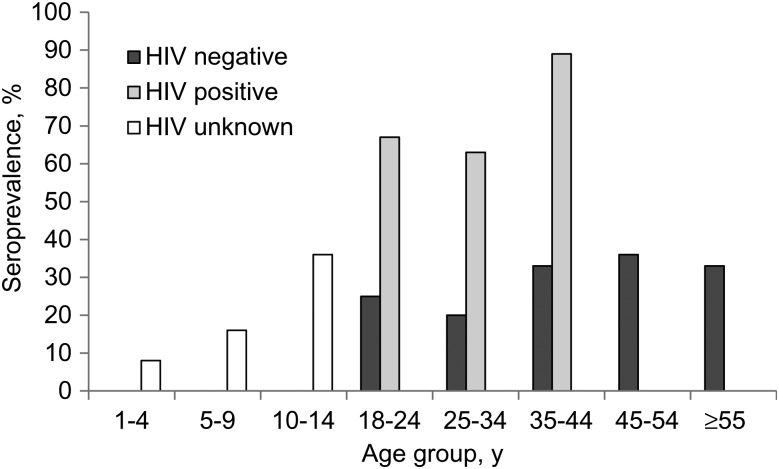
Seroprevalence by age in children (human immunodeficiency virus [HIV] status not tested) and adults (by HIV status) from Misisi compound, Lusaka.

Altogether, 106 serum samples from adults (age range, 18–64 years) and 194 serum samples from children aged 1–14 years were available for analysis. Demographic and clinical characteristics of the children are shown in Table 1 and adults in Table 2. None of the children or adults were jaundiced at the time of blood collection, and none of the IgG-positive serum samples from children or adults were positive for IgM antibodies to HEV. The overall seroprevalence (IgG) in adults was 42% and the overall prevalence in children was 16%, although this varied with age (Table 3). Within the adults, seroprevalence did not differ significantly by age stratum (Table 3). However, within the adult group, in which HIV status was known, HIV had a dramatic impact on HEV seroprevalence (Figure 1). The majority of HIV-seropositive patients had evidence of past HEV infection, irrespective of CD4 count (Table 4). In children, HIV status was not ascertained.

**Table 2.** Demographic and Clinical Characteristics of Adults

Variable	Adults (n = 106)	OR (95% CI)	*P* Value
Sex, M/F, No.	43/63		
Female sex		0.82 (.57–1.2)	.30
Age, y, median (IQR)^a^	33 (25–42)		
Educational attainment^a^ (none/primary/secondary/tertiary), No.	8/66/22/2		
Primary education or none		0.92 (.73–1.2)	.63
Upbringing in urban area up to age 15 y^a^, No. (%)	42 (42)	1.42 (1.0–2.0)	.06
BMI, kg/m^2^, median (IQR)	20.2 (18.7–22.6)		
BMI <18.5 kg/m^2^, median (IQR)		1.21 (.58–2.5)	.63
Hemoglobin, g/dL	14.0 (13.1–15.0)		
Anemia (Hb <12 g/dL)		2.72 (.88–8.4)	.11
Gut parasite infections^a^, No. (%)			
*Ascaris lumbricoides*	17 (17)	0.31 (.1–1.0)	.06
Hookworm	7 (7)	1.93 (.46–8.2)	.44
*Giardia intestinalis*	1 (1)	…	
*Cryptosporidium* species	2 (2)	…	
*Isospora belli*	1 (1)	…	
*Entamoeba coli*	49 (49)	1.09 (.73–1.6)	.84

The OR shown is for risk of hepatitis E virus seropositivity. *P* is by Fisher exact test.

Abbreviations: BMI, body mass index; CI, confidence interval; Hb, hemoglobin; IQR, interquartile range; OR, odds ratio.

^a^ Data only for 194 children.

**Table 3.** Prevalence of Anti–Hepatitis E Virus Immunoglobulin G Antibodies by Sex and Age Group

Age Group	No.	Total	Male	Female
Children				
1–4 y	96	8 (8)	5/42 (12)	3/54 (6)
5–9 y	62	10 (16)	4/22 (18)	6/40 (15)
10–14 y	36	13 (36)	7/15 (47)	6/21 (29)
Adults				
18–24 y	21	8 (38)	3/5	5/16
25–34 y	39	15 (39)	3/12	12/27
35–44 y	25	13 (52)	8/10	5/15
45–54 y	12	4 (33)	3/9	1/3
≥55 y	9	3 (33)	3/7	0/2

Data are presented as No. (%).

**Table 4.** Association Between Hepatitis E Virus Serology and HIV Serology

HIV status	HEV Seropositive	HEV Seronegative
HIV seropositive	22	9
With CD4 count ≥200 cells/µL	12	8
With CD4 count <200 cells/µL	6	1
HIV seronegative	19	48

Odds ratio for effect of HIV on HEV was 6.2 (95% confidence interval, 2.2–17.8), *P* = .0001. HIV testing was only consented to by 98 participants, and CD4 counts were only available for 27 of 31 HIV seropositive patients.

Abbreviations: HEV, hepatitis E virus; HIV, human immunodeficiency virus.

In univariate analysis of risk factors in children, only borderline associations were observed. Storage of drinking water in a closed container was associated with reduced odds of HEV seropositivity (OR, 0.39; 95% CI, .11–1.11; *P* = .06). HEV seropositivity was more common in boys (20%) than in girls (13%). This was not significant (*P* = .17), but sex was used to adjust the odds ratio (Mantel–Haenszel OR, 0.35; 95% CI, .12–1.02; *P* = .045). In unconditional logistic regression, sex, water storage, reported chlorination of drinking water, exclusive use of a pit latrine, and availability of soap in the home were not associated with seropositivity.

In univariate analysis of risk factors in adults, sex was nonsignificantly associated with HEV status, with 49% prevalence in men and 37% in women (*P* = .08). Neither body mass index, mid-upper arm circumference, vitamin A status, or hygiene score below the median were associated with HEV serology. HIV status was strongly associated with HEV serology (Table 4). In the final logistic regression model, female sex was protective against HEV (OR, 0.42; 95% CI, .16–1.08; *P* = .07) and HIV was more strongly associated (OR 8.4; 95% CI, 3.0–23; *P* = .0001).

The relationship between HEV and environmental enteropathy was explored (Table 5). Villous height was lower and crypt depth higher in HEV-seropositive adults. Although epithelial surface area was not significantly lower, there was a consistent pattern suggesting that HEV is associated with more severe environmental enteropathy. HIV interacts with both HEV seropositivity and measures of enteropathy. Villous height was lower in HIV-seropositive (median, 242 µm; interquartile range [IQR], 225–274) than in HIV-seronegative adults (median, 277 µm; IQR, 236–307; *P* = .04). Crypt depth was higher in HIV-seropositive (median, 169 µm; IQR, 159–189) than in HIV-seronegative adults (median, 141 µm; IQR, 130–156; *P* = .0001). When analyzing the effect of HEV on enteropathy separately by HIV group, and vice versa, it appears that HIV has a stronger independent effect on crypt depth than does HEV. There was no association between helminths and enteropathy in this study.

**Table 5.** Mucosal Morphometric Parameters in Relation to Hepatitis E Virus Seropositivity in Adults

Parameter	HEV Seropositive	HEV Seronegative	*P* Value
Villous height, µm	252 (225–280)	272 (237–311)	.05
Crypt depth, µm	160 (141–181)	143 (129–161)	.005
Epithelial surface area, µm	441 (375–544)	478 (404–569)	.24

Data are presented as median (interquartile range).

Abbreviation: HEV, hepatitis E virus.

## DISCUSSION

Hepatitis E virus is a major cause of liver disease worldwide, and although long-term sequelae are rare, the disease carries appreciable mortality in pregnant women [1]. Its relationship to HIV has not received much attention thus far, although several case reports indicate that the clinical picture can be worsened and prolonged in HIV-infected patients [6]. Our data suggest that HEV infection is acquired in Zambia throughout the first 2 decades of life and then the prevalence remains almost unchanged throughout the adult years. The seroprevalence of HEV in this population is higher than in other populations in sub-Saharan Africa: zero in antenatal women in Tanzania [13], 24% of young adults in the Central African Republic [14], 14% of adults in Burundi [15], and 3% in Mozambican refugees in Swaziland [16]. It is also higher than that of adults in Iran (9%) despite our using a more stringent cutoff [17]. Seroprevalence of HEV never exceeds 90% as can happen with hepatitis A virus, [2, 17], probably due to the shorter duration of the IgG response, which was measured at a half-life of 14 years following an outbreak of HEV in Kashmir [18]. Most HEV infections are asymptomatic, especially in children [2]. We found a modest and nonsignificant increased prevalence in males, both adults and children, which is consistent with the higher attack rate in men in most epidemics [2]. In our study, prevalence did not change in adult age groups, and there was no suggestion of an age cohort effect as seen in Iran [17].

A decade elapsed between the sample collection for the adults (2001) and the children (2011), which is a consequence of our decision to use serum samples from a study of environmental enteropathy. Although this might have allowed secular changes to alter the HEV transmission profile in this community, we have seen no evidence of major changes in drinking water or sanitation, and the *Helicobacter pylori* seroprevalence in adults has remained absolutely constant at 81% [19, 20].

HIV was strongly associated with HEV status in adults. We did not test the children, but we know from other work that HIV seroprevalence in children in this community is approximately 5%. The association in adults signifies either that HIV induces a greater likelihood of serological detection of HEV exposure (eg, through the well-known polyclonal hypergammaglobulinemia), or that it increases susceptibility to HEV infection, or that it increases the longevity of the IgG response. We suspect that increased susceptibility is the likeliest explanation. There was a suggestion that serum samples positive for HEV were more likely to come from patients with CD4 counts <200 cells/µL, but our sample size would have to be higher to ascertain with confidence whether HEV risk increases as CD4 count falls. French data did not demonstrate a relationship between HEV seropositivity and CD4 count [21], but Swiss data suggested the opposite [22]. It is clear that more work needs to be done on this question. There are few data on HEV infection and HIV in Africa, but there is evidence that coinfected pregnant women have higher HIV RNA load [23].

The etiology of tropical enteropathy has long been an enigma, but through the weight of accumulating evidence that environmental factors play a dominant role, rather than latitude per se [9], it is now more accurately referred to as environmental enteropathy [8, 24]. We know that in high-density residential areas in and around Lusaka, there is coliform bacterial contamination of drinking water [12]. This contamination is worse in household stored water than in source water, suggesting direct fecal contamination on hands or drinking vessels introduced into stored water. The data presented here suggest that poor-quality drinking water does increase HEV transmission in children. There is no evidence that HEV infects epithelial cells in the intestine. We consider it to be more likely that HEV seropositivity is a biomarker of high exposure to intestinal pathogens. However, there are interesting interactions between HIV, HEV, and enteropathy. HEV and HIV are both associated with similar changes in villous height and crypt depth, and both are associated with nonsignificant reductions in epithelial surface area. When subgroup analysis is done, HIV has an independent effect on crypt depth, and HEV has no effect on crypt depth in subgroups divided by HIV status. This is not true for villous height, and it is likely that larger studies may be required to resolve the relative contributions of these 2 viruses. We know from previous work in this community that the dominant effect of HIV on the mucosa is increased crypt depth, and this was observed in the present study also. These samples were collected from the period before combination antiretroviral therapy (cART) was widely available. It would be interesting to know if the association between enteropathy and HEV still holds true in the era of cART.
